# From Doubt to Direction: Untangling Pediatric Scrupulosity

**DOI:** 10.3390/children12040528

**Published:** 2025-04-21

**Authors:** Rachel E. Mathews, Shivali Sarawgi

**Affiliations:** 1 Cincinnati Children’s Hospital Medical Center, Cincinnati, OH 45229, USA; 2 College of Medicine, University of Cincinnati, Cincinnati, OH 45221, USA

**Keywords:** obsessive-compulsive disorder, pediatric OCD, scrupulosity, religiosity, moral OCD, values, distress tolerance, exposure with response prevention, intolerance of uncertainty

## Abstract

**Background**: Up to 33% of individuals with obsessive-compulsive disorder (OCD) have scrupulosity symptoms, although less is known regarding the prevalence rates in youth, specifically. Scrupulosity translates to “fearing sin where there is none” and describes pathological guilt and distress related to religion and morality. Disentangling scrupulosity from true religious beliefs and actions may be difficult in youth for a number of reasons, including the nature of youth as a time of developing independent identities and values, expected ritualistic behavior (e.g., confession, ritualistic cleansing), scrupulosity being reinforced in some religious communities, and the discomfort or inexperience of clinicians with both these symptoms and various belief systems. The literature suggests limited knowledge of scrupulosity among mental health providers, including pediatric clinicians, and apprehension to discuss or target scrupulous beliefs and behaviors. Apprehension may be enhanced for providers working with youth populations, particularly given broader misconceptions about the efficacy and safety of gold-standard interventions. **Objectives**: This narrative review with practice guidelines examines the existing literature related to pediatric scrupulosity and its challenges and describes evidence-based treatments for scrupulosity in pediatric populations. Recommendations for clinical practice and research are discussed.

## 1. Introduction

Obsessive-compulsive disorder (OCD) is defined by the *Diagnostic and Statistical Manual* (DSM-5-TR) as the presence of obsessions (e.g., contamination, harm), which cause distress and are experienced as intrusive, as well as repetitive behaviors (e.g., cleaning/washing, checking). These symptoms are time-consuming, significantly distressing, and functionally impairing [1]. OCD affects approximately 2% of the adult population and approximately 1–2% of pediatric populations [2]. There are several OCD subtypes, which include, but are not limited to, contamination, aggression/harm symptoms, sexual obsessions, symmetry or “just right” symptoms, and scrupulosity. Existing research suggests that contamination OCD and checking compulsions are the most common OCD subtypes [3]. As such, there have been numerous studies examining these subtypes, resulting in “mini models” of conceptualization and treatment [4]. Within the pediatric population, research by Selles, Storch, and Lewin [5] found that younger children demonstrated increased hoarding compulsions, while older youth were more likely to experience sexual obsessions, magical thinking and somatic obsessions, with accompanying checking, counting, and magical thinking rituals. However, there is scant literature examining scrupulosity in youth [6], as well as limited data examining the interaction of scrupulosity with other OCD subtypes (e.g., cleansing, just-right symptoms related to spirituality).

Scrupulosity translates to “fearing sin where there is none” [7]. More broadly, scrupulosity is an OCD presentation that consists of excessive religious and/or moral fears [8,9] and may also include fear of taboo thoughts. Other elements of scrupulosity involve excessive fears of immorality or committing a sin, intrusive blasphemous thoughts, excessive religious practices [10,11], reassurance-seeking [7], and other types of “ethical rightness or perfection” [12]. Common religious obsessions often include worries about committing a sin (e.g., lying), fears of damnation or demonic possession, concerns about performing religious practices (e.g., praying) imperfectly, obsessional thoughts about the Devil, and worries about being insufficiently devoted to one’s religious beliefs [10,11,13]. Common religious compulsions may involve excessive praying, excessive confession, reading/rereading religious literature, redoing religious tasks (often until they are completed “perfectly” or feel just right), and excessive reassurance-seeking from religious leaders or loved ones [7]. An early scrupulosity study by Foa et al. [14] identified religious beliefs as the fifth most common OCD theme, with nearly 6% of OCD patients reporting scrupulosity as a primary obsessional symptom. The prevalence rates of scrupulosity within OCD patients range from 5% to 33% of patients [9].

Scrupulosity is associated with poorer insight [15] and increased internalizing symptoms [13], potentially complicating treatment and necessitating increased attention, particularly for pediatric populations. Since one of the earliest publications focused on describing scrupulosity in pediatric populations [16], few studies have examined treatment or provided specific recommendations in addressing this symptom dimension in youth, which may be especially challenging due to the influence of family and peers as well as normative identity development. Below, we present a narrative review of the limited literature specific to treating scrupulosity symptoms in youth as well as targeted practice recommendations.

## 2. Common Challenges Associated with Scrupulosity

To date, there has been limited research examining the evaluation and treatment of scrupulosity in youth and adolescents. However, in adult populations, identifying and treating scrupulosity remains an area in which providers continually express apprehension. The existing literature suggests there is limited knowledge of this symptom presentation among mental health providers [17,18]. This may be further complicated by a tendency among providers to forego the implementation of exposure-based treatments in OCD patients due to personal discomfort [19], despite exposure with response prevention (E/RP) being the gold-standard treatment. In the case of scrupulosity, providers may also be more apprehensive to implement E/RP due to uncertainty in disentangling religious beliefs from compulsions, as well as the risk of offending, questioning, or dissenting with the patient and/or family’s belief system or values. Members of religious communities may also unintentionally reinforce pathological religious rituals through normative practices or provide treatment-interfering reassurance due to limited awareness of OCD and its presentation, such as Catholic confession, Ramadan fasting, and Amrit Sanskar [18,20]. Thus, the treatment of scrupulosity, whether with pediatric or adult populations, requires careful consideration specifically because many religious rituals lend themselves well to OCD presentations. For instance, praying, ritualized bathing, and confession are widespread religious customs but are also considered common compulsions within the context of OCD. Additionally, religious OCD can be easily reinforced because OCD often takes a “more is better” approach (e.g., “if my belief system recommends that I pray three times daily, six times would be even ‘better’”). Indeed, a study by Siev, Baer, and Minchiello [21] identified that approximately 20% of participants with scrupulosity reported beliefs that their scrupulous symptoms enhanced their religious experiences.

The extant literature supports the adverse impact of scrupulosity on patients’ reported religious relationships. Research by Nelson, Abramowitz, Whiteside, and Deacon [13] found that individuals with scrupulosity often attend more to minor details of their religious beliefs, at the expense of more pertinent or critical areas. Additionally, a study by Siev, Baer, and Minchiello [21] found that approximately 70% of patients with scrupulosity endorsed interference in their religious observance or relationship with God/a Higher Power. This same study determined that approximately 32% of patients with non-scrupulous OCD reported such disruption. Increased scrupulosity has also been found to be associated with a more negative perception of God/a Higher Power, seeing such an entity as punishing or vengeful [21]. However, it is presently unclear whether a negative concept of God is either a risk factor or a result of pathological religious distress.

The belief that symptoms of scrupulosity improve one’s religious experiences may further complicate the already muddy distinction between normative religious beliefs and religious/moral OCD. The degree of functional impairment resulting from one’s spiritual beliefs and practices can aid in discerning the difference between these concepts. More specifically, if an individual feels that they must engage in religious rituals in order to address one or more of the three core motivators of OCD (disgust, incompleteness, and/or harm avoidance) [22], then scrupulosity OCD may be a more likely factor. Ego-dystonicity is another factor that ought to be considered when attempting to disentangle scrupulosity from typical religious practices and/or moral beliefs. Ego-dystonic thought has been described by Purdon et al. [23] as “…one that is perceived as having little or no context within one’s own sense of self or personality. That is, the thought is perceived, at least initially, as occurring outside the context of one’s morals, attitudes, beliefs, preferences, past behaviors, and/or one’s expectations about the kinds of thoughts one would or should experience” (p. 200). This may be characterized by an undercurrent of fear or distress/discomfort associated with religious/moral beliefs and behaviors versus wanting to have a relationship with one’s God/Higher Power or growth mindset related to one’s character. Likewise, scrupulosity may be at play if the individual is engaging in religious rituals that are well outside the bounds of what is considered typical practice by their belief system in terms of time, intensity, and/or duration.

Of note, holding religious beliefs and engaging in faith-based practices is not inherently deleterious—on the contrary, there is an abundance of literature substantiating the positive utility of spiritual beliefs and customs [24]. More specifically, personal faith systems have been shown to aid in adjustment and coping related to a range of life events (including, but not limited to, death and chronic illness), sense of community and social support, and identity development [25]. However, in individuals with scrupulosity, it is important to consider how liturgical beliefs and participation in related practices may interact with their OCD in a manner that results in excessive distress and functional impairment. Thus, mental health providers should collaborate with faith leaders to encourage utilization of exposure-based treatment in a way that honors the patient’s religious values while extinguishing OCD symptoms. A key indicator for the presence of OCD versus normative religious beliefs or practices is the interference (or lack thereof) related to symptoms. If an individual’s symptoms are interfering with functioning (such as over-focusing on minor details or “going through the motions”, rather than promoting spirituality and faith value), this may be an indication that OCD is influencing the individual’s spiritual thoughts and related behaviors.

**Figure 1:**
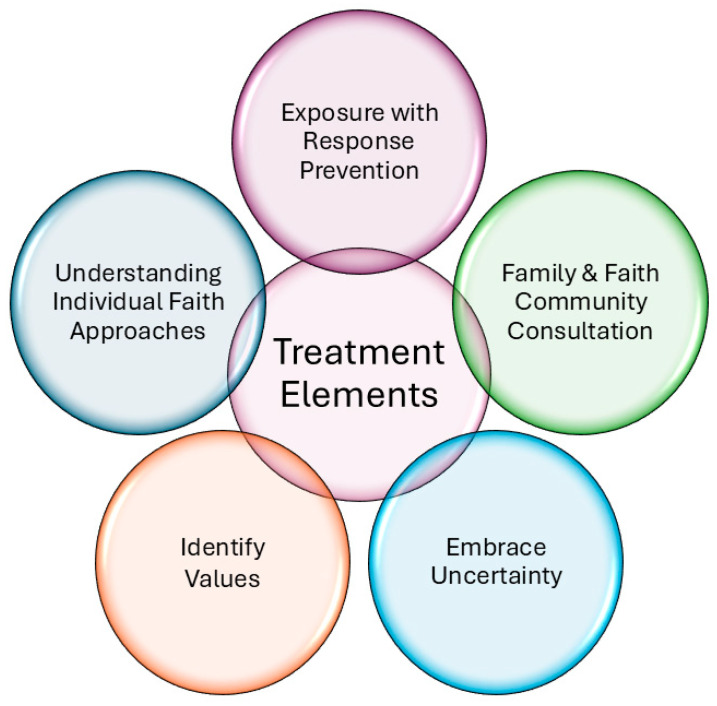
Key elements in scrupulosity treatment.

For example, prayer is a ubiquitous spiritual practice that is a key component in most faith-based systems, and the act of engaging in prayer is often strongly encouraged as part of many religious customs [26]. For individuals without OCD, praying to a God/Higher Power is often reported to be a positive experience that strengthens existing spiritual beliefs, provides comfort, and enriches one’s relationship with a God/Higher Power [27]. However, for individuals with OCD, they may feel the need to pray more frequently than is recommended by their religious organization, for longer durations than are expected, or repetitively if they feel the prayer is not “perfect” or said in a specific order in order to alleviate feelings of distress—thereby transforming what is often viewed as a helpful religious custom into a distressing and demanding set of rules. Below, we discuss key elements of treatment (see Figure 1) to address the aforementioned concerns.

## 3. Evidence-Based Strategies

### 3.1. Assessing Typical Versus Excessive Faith and Moral Practices

Rituals, whether religious or secular, are found in virtually all beliefs systems around the world [28]. The purpose of rituals varies among cultures and/or belief systems but may be carried out to establish a divine connection with a Higher Power, provide a sense of community, and create a sense of order within a given society. Rituals may involve actions/behaviors, words, gestures, or objects, and tend to be performed within a specific sequence [29,30]. Common types of rituals include, but are not limited to, praying, cleansing, sacraments, rites of passage, rites of intensification, and rites of affliction [31]. Interestingly, it should be noted that the term “ritual” is often used interchangeably with the term “compulsion” within the context of OCD diagnosis and treatment. However, when treating scrupulosity, providers must distinguish between a religious ritual (whose purpose is to enhance one’s existence and provide meaning) versus an obsessive-compulsive ritual, which is ego-dystonic, distressing, and interferes with values-based living and decision-making. This can be a challenging undertaking with youth, who may possess limited insight regarding the excessiveness of their distress related to religious worries, as well as the frequency, duration, and/or intensity with which they are performing religious rituals. Thus, varying degrees of insight within the pediatric population can complicate the process of teasing apart normative religious rituals from scrupulosity OCD. As such, engaging other members within the child’s family and religious system is an essential component of assessing and treating pediatric scrupulosity.

Because different belief systems, communities, and family systems retain different norms, it is important for clinicians to familiarize themselves with the key principles of such systems when they begin working with patients with scrupulosity. This may be accomplished through ongoing discussions with both the patient and family members. There may be variations in religious practices at different system levels, necessitating further consultation with the family to ascertain which behaviors are deemed inappropriate or excessive. For instance, a family who follows the Catholic faith may say grace before meals and engage in evening prayer but forego morning prayer and Liturgy of the Hours. As such, it is necessary to have both a broad understanding of standard religious customs within a belief system and insight into family practices within the context of faith. Furthermore, ongoing consultation with caregivers is necessary, as patient perceptions of their OCD presentation may be impacted by their age/developmental level and/or insight into their symptoms. Of further importance is considering the role of normative development in forming specific moral or religious behaviors. One major task of adolescence is experimenting with and forming one’s own identity. As such, it would be considered developmentally appropriate for adolescents to trial various aspects of their appearance, attitudes, and beliefs, which may include religious or moral ideologies. An adolescent’s experimentation with religious and/moral identity is not an inherent cause for concern; however, it may become concerning if the individual experiences functional impairment and/or excessive, impairing distress as a result of their religious/moral identity exploration. It is also crucial for mental health providers to collaborate with caregivers for parent training and to ensure caregivers are not unintentionally reinforcing symptoms of scrupulosity [4].

Likewise, it may be beneficial to consult with religious leaders associated with specific belief systems for additional clarification on the appropriateness of religious behaviors [32,33,34,35]. This collaboration may assist in shedding light upon the faith values of a belief system—in other words, what is considered a “typical” component of a religious belief system versus what may be considered excessive or unusual for that particular system. Consulting with faith leaders may aid in clarifying specific aspects of faith that may be ambiguous or uncertain from a theological standpoint versus through an OCD lens. For example, within the Islamic faith, Salat (ritual prayer) is a key practice and includes four different types of ritual practice (*Fard Salat*, *Wajib Salat*, *Sunnah Salat*, and *Nafl Salat*) [36], which are performed at designated times each day. Similarly, within the Jewish faith, morning prayer (*Shacharit*), afternoon prayer (*Minchah* or *Mincha*), and evening prayer (*Ma’ariv* or *Arvit*) are considered standard, daily religious practices [37,38,39]. It should also be noted that certain practical beliefs and related compulsive behaviors may be influenced by particular recommendations from a faith leader and/or family structures. For example, if one’s pandit is intensely focused on repeating specific mantras at temple, this may be considered excessive within the structure of the broader Hindu faith and may unintentionally interact with just right/incompleteness OCD. Likewise, if a family tends to apologize excessively for perceived errors, this may shape disproportionate over-apologizing behaviors in youth with OCD (see Appendix A). Thus, when working with individuals who are operating within different belief systems, it is essential for mental health providers to engage in continuing collaboration with spiritual leaders, as this will allow the patient to practice their faith-based beliefs within the bounds of their chosen belief system.

### 3.2. Exposure with Response Prevention (E/RP)

Although scrupulosity can be a complex and nuanced presentation of OCD, it is encouraging to know that the treatment aligns with the gold standard treatment for OCD as a whole: exposure with response prevention (E/RP) [40,41]. Within the context of scrupulosity, development of the fear hierarchy is an especially critical step, as this process is essential in distinguishing religious standards of practice versus excessive, impairing compulsions. As previously discussed, it is essential for mental health providers to collaborate with parent(s)/caregiver(s) to ascertain which behaviors are or are not excessive [9] so that an appropriate hierarchy can be developed and executed. When building a hierarchy for youth with scrupulosity, it will likely be helpful to utilize more concrete rather than abstract strategies, particularly when working with younger children. Additionally, engaging in exposures that will help build general distress tolerance and tolerance of uncertainty will be beneficial for youth with scrupulosity, as this may aid in generalization of skills. Including value-based and distress tolerance-focused elements in E/RP (as described in more detail below) may also be beneficial. Likewise, engaging in exposures with the patient whenever possible has been shown to be efficacious in both symptom management and long-term treatment outcomes. This is another area in which the mental health provider ought to consider their own religious beliefs (or lack thereof) and how these viewpoints may impact the implementation of exposures and patient perceptions of treatment as a whole.

It should be noted that when treating OCD broadly, the development of a strong therapeutic alliance is paramount. The degree of trust required of the mental health provider when guiding an individual through exposure-based treatment necessitates ample rapport-building from a general perspective as well as within the context of E/RP. Furthermore, the deeply personal nature of scrupulosity is a delicate matter that requires comprehensive understanding on the part of the provider in terms of both normative religious beliefs and fears held by the patient. One method of building rapport, particularly in youth with scrupulosity, is having transparent and often vulnerable discussions related to intrusive religious/moral thoughts, ritualized behaviors that are deemed excessive, and the interplay of these symptoms within the bounds of normative practices within their belief system. These conversations can be strengthened through self-disclosure from the mental health provider, when appropriate. For example, it may be beneficial for the provider to identify similarities between the provider’s beliefs and those of the patient, as this can reinforce understanding and relatability with the provider. Likewise, acknowledging differences in the beliefs of the provider and patient may also bolster the therapeutic alliance, as this process may allow the provider to reiterate that the patient is enduring a unique lived experience and help guide decision-making for treatment goals. In sum, it is essential that the provider maintain an open and non-judgmental demeanor versus an attitude that is minimizing or dismissive. This is a scenario where the use of humor, which can have its place in E/RP, may not be appropriate. An overly dismissive or humorous approach in this situation may leave the patient feeling ashamed and foolish, which may adversely impact treatment (or even the patient’s willingness to return to the clinic for ongoing care).

### 3.3. Values-Based Treatment Approaches

As is often the case with OCD, scrupulosity attaches itself to a subject that is of high meaning and value to the individual with OCD, such that related distress is magnified by the subjective weight placed on the obsession or compulsions. Impairment associated with scrupulosity is similar to that of other OCD presentations, such that time and energy dedicated to obsessive thinking and execution of compulsions impact relationships, interfere with focus/attention, and move patients further away from values, in contrast to the very motivations behind OC symptoms. As such, scrupulosity can be difficult to manage when having a relationship with a Higher Power is of value to the individual with OCD. Values have been described by Twohig et al. [42] as providing direction to life, guiding one’s actions, rather than being concrete, actionable goals. Wetterneck, Lee, Smith, and Hart [43] elaborated, “In addition values are never achieved or completed; rather they provide meaning and direction for behavior. If goals are a destination, then values are a direction” (p. 68). A focus on values can further ensure the functionality of treatment goals and individual exposures within E/RP as opposed to taking a “fear factor” approach. This is of particular importance when addressing scrupulosity in youth, as E/RP without values-based meaning may contribute to overestimation of guilt and uncertainty without building healthy tolerance. This is the difference between formulating an exposure of praying in a new, non-ritualized manner versus asking a patient to pray to something counter to their beliefs (e.g., for patients praying according to the Lord’s Prayer, praying conversationally versus praying to the universe). Values-based E/RP has been shown to increase motivation [44], psychological/cognitive flexibility [45], and quality of life [46], particularly from the perspective of Acceptance and Commitment Therapy (ACT) when incorporating ACT-based strategies. ACT is considered a mindfulness-based behavioral or third-wave cognitive-behavioral therapeutic approach that promotes a healthier relationship with one’s internal experiences. E/RP from this perspective places an emphasis on exposure to distressing stimuli, including triggers for scrupulous intrusive thoughts, as a means of approaching values and increasing fulfillment in life over habituating to specific stimuli [42,47]. It encourages acceptance of these triggered intrusive thoughts and related distress without attempts to control or change these (e.g., through compulsions). Similarly, it encourages patients to spend less time trying to understand or change their intrusive thoughts, which allows more time for values-based living.

Given that identity, insight, and abstraction are still developing processes in youth with OCD, values may be unclear or little-thought-of concepts. Therefore, it is important to first help individuals identify and define values, with an expectation of potential change in some values throughout development. Defining personal values may help patients differentiate between faith-based actions and OC-driven behavior or focus on core experiences of faith. However, it can be difficult for youth to distinguish values from OC-driven motivations. As described above, inclusion of patients’ families in scrupulosity treatment can be essential, particularly given the reciprocal nature of faith and religious beliefs between caregiver and child [4]. The family’s values can also serve as more objective indicators of faith-based practices rather than those influenced by OCD, creating a healthier point of comparison. This is particularly relevant given the previously discussed uncertainty or ambiguity found in faith and religion. Given that family values are not always in line with a patient’s values and that a patient’s spiritual identity may be different from their caregivers’, we have suggested the involvement of faith leaders and spiritual communities in treatment (see above). Understanding the values of a patient’s spiritual, faith, and broader social communities can further help level set for the patient without having to rely on regular checking-in that may develop into or increase reassurance. Given the values orientation, ACT-based approaches to E/RP suggest a focus on engaging in meaningful, values-directed actions as the exposure regardless of internal experience. At the same time, it is important to encourage patients to refrain from attributing meaning to these internal experiences during an exposure, which may be more ambiguous and easily warped by OCD (e.g., an individual attaches labels of spiritual conviction or moral wrongness to internal experiences of distress). As such, assisting patients in identifying which behaviors are values-based can help prevent them from falling into these traps, which can become circular and/or increasingly impairing. Values should, therefore, be both identified prior to beginning E/RP and continuously assessed.

### 3.4. Targeting Uncertainty

A well-established etiological and maintaining factor in OCD is intolerance of uncertainty (IU). IU is a transdiagnostic factor indicating cognitive, behavioral, and emotional distress and difficulty tolerating the unknown [48]. Indeed, IU has been identified, by some, to be central to the experience and presence of scrupulosity [49]. Spiritual uncertainty and doubt are also thought to increase with age, with late adolescence being a time of particular religious uncertainty [50]. This is perhaps due in part to adolescence being a necessary period of identity development, as discussed above. While most belief systems espouse a clear ideology, with set rules, commandments, and/or expectations, the nature of faith itself (whether it be related to religious or secular beliefs) can leave room for doubt. General convictions of conscience and ideas of morality, even for those without a specified faith, are equally susceptible to uncertainty. Uncertainty, therefore, appears inherent in most major religious belief systems and spiritual practices—perhaps, intrinsically linked to the proliferation of various theologies and interpretations of religious texts and spiritual practices. Frankel [51] describes a process of learning to “bear” uncertainty, suggesting, “The unknown is a deep current that runs throughout all religions and mystical traditions, and it is also the nexus of contemporary psychotherapeutic thought and practice”. Research by Greenberg, Witzum, and Pisante [52] further suggested that IU in OCD patients may contribute to difficulties in distinguishing normal religious practices from OC symptoms, a concern addressed above. The desire for a clear and finite answer is one element of scrupulosity that can be particularly frustrating for patients experiencing such symptoms and must be considered during the treatment process. Rituals may appear particularly appealing to patients with scrupulosity because they serve to reduce anxiety and uncertainty, provide a sense of control [53,54], and counter mental disturbance [55]. Moreover, higher levels of IU have been linked to decreased treatment effects on anxiety and functional impairment for pediatric OCD populations; optimistically, a greater reduction in IU was associated with improved treatment gains on related anxiety and functionality [56]. Tolerating uncertainty has been demonstrated to be an efficacious intervention target in OCD across the lifespan and may, given these past findings, be of particular importance in treating scrupulosity in pediatric populations. This can be targeted through E/RP that evokes and maintains uncertainty, especially when paired with an ACT approach (as described above). For example, touching an object that may or may not be contaminated and preventing a compulsion to increase certainty (e.g., asking a parent if it was actually dirty) or mitigate risk of harm (e.g., washing hands just in case). The following tools may be beneficial for addressing uncertainty related to scrupulosity, specifically.

Youth may feel particularly reluctant to tolerate uncertainty related to spiritual or moral matters, especially in the context of E/RP. Expectancy violations (i.e., when the outcome is contrary to what OCD predicted) are a main goal in E/RP, according to the inhibitory learning model [57]). Expectancy violations for scrupulosity symptoms may not yield data in the near future, or it may be considered “too late” or “too risky” by the time youth feel as if they will know whether what they did was “right” or “wrong”. For example, a scrupulosity exposure may include reading scripture at a different time of day or frequency, not expressly forbidden by the faith; for a patient with scrupulosity, the feared consequence of such an exposure may be damnation or eternal consequences that will not be known until it is “too late”. However, shifting the focus of this exposure to building uncertainty tolerance may prove beneficial, and prior uncertainty tolerance exposures may set the stage for more success with scrupulosity-focused E/RP that are naturally more uncertain. In another example, an adolescent may feel reluctant to decline a request for help by a peer, even for objectively important reasons, due to fear that not helping may make her a “bad” person or may lead to a misfortune for her. In linguistic processing of the exposure and in consolidating learning, a provider should reinforce the ability to engage in the exposure and/or to sit in the uncertainty, despite a level of moral ambiguity or perceived potential for harm.

Historically, habituating to a stimulus or action was a primary goal of E/RP. However, this does not address the experience some youth may have that it feels “wrong” to habituate to scrupulosity-related distress. That is, engaging in behavior counter to what OCD may lead one to believe is necessary to their faith or morals and not feeling distressed may be a sign of moral decay or lack of commitment to a faith. For religious symptoms, specifically, this may be related to ideas that scrupulosity allows one to connect more completely with one’s religion. Here again, shifting the focus to tolerance of the uncertainty-related distress, versus habituating to “being bad” or sacrilegious, both honors a patient’s faith and provides a more generalizable inhibitory learning. Efforts to “boss back” OCD with reassurance and theological arguments (e.g., “I am allowed to pray at different times”), as opposed to evidence and distress tolerance developed during E/RP, are shown to be minimally effective in reducing and managing scrupulosity symptoms long-term. Reassurance and theological arguments from patients themselves, or others may be more susceptible to the increased doubt inherent in OCD, particularly because uncertainty will naturally remain in these spiritual concerns. For example, a patient may experience uncertainty about the occurrence of karma due to perceived hurtful or sinful behavior; when seeking reassurance about this, it is appropriate to reinforce acceptance of the unknown and uncertain nature of faith as well as within the context of OCD. Fortunately, approaching E/RP from the lens of building distress/uncertainty tolerance [57,58] and targeting core motivations [22], regardless of surface-level symptoms, can set the stage for E/RP with scrupulosity symptoms. It is beneficial to focus on these underlying factors when engaging the patient in processing and learning consolidation post-exposure, even for thematically different symptoms. This can further help ensure that youth are entering into scrupulosity exposures with the right mindset. It may also help foster necessary insight for the patient regarding the driving force behind certain thoughts and rituals (i.e., fear or intolerance of uncertainty versus conviction and faith or conscience). It is therefore essential for mental health providers to continue pushing on and highlight the uncertainty for patients when fear is at play. However, in highlighting uncertainty, there is no need to encourage doubt in the patient’s faith or moral system itself; rather, bring attention to the inherent uncertainties in the faith through utilization of the above tools to target functionality. According to guidance from family, spiritual leaders, and an individual’s faith community, increase functional spiritual practice as well as engagement in values (see above) where uncertainty may still remain. Embracing uncertainty in scrupulosity, particularly with an ACT lens, further provides the necessary space for identity development and exploration, which is developmentally appropriate for this age group.

## 4. Discussion

There are numerous challenges in treating pediatric scrupulosity, as it can be difficult to disentangle religious beliefs and/or behaviors from religious compulsions. Likewise, varying moral beliefs within family units may also further complicate the identification and treatment of scrupulosity. Lack of familiarity with various belief systems, concerns of offending the patient and/or family, and discomfort in completing exposure-based treatment may all interfere with therapeutic progress and, thereby, contribute to poor or limited treatment outcomes when addressing scrupulosity within the clinical setting. Nevertheless, E/RP remains the gold standard treatment for scrupulosity and should be implemented with the same frequency and intensity as non-religious exposures. Values-based treatment and provision of distress tolerance skills are also beneficial in supplementing traditional OCD treatment for scrupulosity. The process of treating scrupulosity will likely require the clinician to partner closely with the patient and family members, as well as religious leaders outside of the family unit.

## 5. Future Directions

There are several pertinent directions for future research within the area of pediatric scrupulosity. First, it is essential that scrupulosity with children and adolescents be studied more extensively to better understand the changing landscape of both religious and secular beliefs. More specifically, it will be beneficial for future research to empirically evaluate the key components involved in treating pediatric scrupulosity, including those suggested above, such as E/RP and E/RP + ACT, as has been explored in the adult literature. Furthermore, qualitative research examining thematic elements of scrupulosity in pediatric populations would fill a substantial gap within the existing literature base. Additional directions for future research should also involve ongoing efforts within the field to expand patient access to exposure-based intervention, with an emphasis on the provision of high-quality, rigorous training for providers in navigating spiritual concerns and faith communities. Finally, future research should include ongoing education for both mental health providers and faith communities about the nature of scrupulosity as a presentation of OCD.

## 6. Conclusions

Although there is limited research examining the conceptualization and treatment of scrupulosity OCD in pediatric populations, there is a well-established literature base supporting the utility of E/RP in treating OCD. The emphasis of E/RP on both building distress tolerance and aiding in long-term symptom management so that individuals with OCD may experience greater functioning and quality of life is integral to its efficacy. In particular, scrupulosity offers unique challenges to the already idiosyncratic nature of OCD. Although scrupulosity is a complex and nuanced OCD presentation, it can be appropriately addressed by mental health providers in pediatric populations through ongoing collaboration with stakeholders and openness to furthering one’s own understanding of varied religious practices.

## Data Availability

No new data were created.

## Abbreviations

The following abbreviations are used in this manuscript:

OCD	Obsessive-compulsive disorder
E/RP	Exposure with response prevention
ACT	Acceptance and commitment therapy
OC	Obsessive-compulsive
IU	Intolerance of uncertainty

## Appendix A

Table of themes and practice recommendations based on extant literature.

**Themes**	**Description**	**Examples**
Exposure with Response Prevention	•Gold-standard intervention to reduce excessive, impairing thoughts and accompanying compulsions associated with religious/moral beliefs deemed excessive to a particular belief system. •Goal of patient practicing within the bounds of their belief system. •Builds distress tolerance and allows for experiential, inhibitory learning.	•Patient adhering to Christian faith feels prayers must be said perfectly and may “start over” if a “bad” or sinful thought intrudes or if the prayer does not feel “right”. Exposure: Praying a new or spontaneous prayer Response prevention: Praying out loud with family who can prevent repeating or starting over •Hindu patient excessively rewashes hands prior to touching an idol in the home temple. Exposure: Turn off the bathroom lights after washing hands and before entering home temple Response prevention: Praying out loud with family who can prevent repeating or starting over
Understanding Individual Faith Approaches	•A provider should familiarize self with a patient’s specific beliefs and a patient-center context. •Identify a patient’s priorities, such as: spiritual connection, joy in faith, salvation, bringing positivity to one’s environment, following up-to-date doctrines, being a good person.	•A female Catholic teenager is interested in pursuing church leadership and begins to align with other Christian approaches that would allow her to lead a church or preach. (Not OCD) •An individual apologizes frequently and is concerned with putting out “good into the world”. This is not due to true character and identity development but out of fear of being “bad” or of something “bad” happening. (OCD, not individual morality)
Family & Faith Community Consultation	•Collaboration with faith leaders for the purpose of gaining insight related to normative practices within a specific belief system. •Building understanding of what would be considered excessive or unusual practice for members of a patient’s family or for most in their community.	•Consulting with an Imam regarding typical cleansing practices or number and frequency of prayers within the context of a specific Muslim faith. •Discussing family rules and teachings with caregivers to understand family culture with ideas such as “doing right” or being “good,” lying, judging others, or being helpful. •Collaborating with a Rabbi to understand prayer rituals and adherence to Sabbath rules for a family’s level of orthodoxy or traditionalism.
Identifying Values	•Understanding and incorporating patient values is crucial for effective E/RP, particularly with scrupulosity. •Emphasizes living in faith or living out one’s morality versus living and doing out of fear. •E/RP allows patients to move toward values and engage in spiritual practices in a non-ritualized manner, without acting contrary to their beliefs/morals. Scrupulosity may actually distance one from their values.	•Exposures in alignment with values: Saying the Lord’s Prayer “correctly” and in its entirety, even if a “mistake” is made Setting a budget and donating to a cause of interest Not eating meat or eating certain vegetarian foods without receiving reassurance that it is safe •Exposures *not* in alignment with values: Saying a satanic prayer Donating more than affordable or fiscally responsible to a cause of interest Intentionally eating something that contains meat
Embracing Uncertainty	•Uncertainty is inherent in both OCD and faith/morality. Building uncertainty and related distress tolerance is a useful target. •Providers can help patients and families identify and accept uncertainty in their beliefs, practices and daily lives.	•Guiding an adolescent through E/RP of declining an ask for help from a peer without reassurance or knowing if it will lead to being a “bad” person or something “bad” happening. •Discussing certain topics of faith or morality as being ambiguous or grey, e.g., is there ever a time when it is “okay” to lie. •Helping a child through an exposure of saying a prayer out of order (in a manner not expressly forbidden by their faith) not knowing if this will anger a Higher Power.

## References

[1] Association A.P. (2022). Diagnostic and Statistical Manual of Mental Disorders.

[2] Rough H.E., Hanna B.S., Gillett C.B., Rosenberg D.R., Gehring W.J., Arnold P.D., Hanna G.L. (2020). Screening for pediatric obsessive–compulsive disorder using the obsessive-compulsive inventory-child version. Child Psychiatry Hum. Dev..

[3] Rowsell M., Francis S.E. (2015). OCD subtypes: Which, if any, are valid?. Clin. Psychol. Sci. Pract..

[4] Abramowitz J.S., Jacoby R.J. (2014). Scrupulosity: A cognitive-behavioral analysis and implications for treatment. J. Obs.-Compuls. Relat. Disord..

[5] Selles R.R., Storch E.A., Lewin A.B. (2014). Variations in symptom prevalence and clinical correlates in younger versus older youth with obsessive–compulsive disorder. Child Psychiatry Hum. Dev..

[6] Stevens S., Smith-Schrandt H.L. (2023). Scrupulosity Obsessive-Compulsive Disorder in Children. J. Psychosoc. Nurs. Ment. Health Serv..

[7] Abramowitz J.S., Buchholz J.L., Rosmarin D.H., Koenig H.G. (2020). Spirituality/religion and obsessive–compulsive-related disorders. Handbook of Spirituality, Religion, and Mental Health.

[8] Pirutinsky S., Siev J., Rosmarin D.H. (2015). Scrupulosity and implicit and explicit beliefs about God. J. Obs.-Compuls. Relat. Disord..

[9] Greenberg D., Huppert J.D. (2010). Scrupulosity: A unique subtype of obsessive-compulsive disorder. Curr. Psychiatry Rep..

[10] Cassiday K.L. (2023). Helping Children and Teens with Difficult-To-Treat OCD: A Guide to Treating Scrupulosity, Existential, Relationship, Harm, and Other OCD Subtypes.

[11] Bonchek A., Greenberg D. (2009). Compulsive prayer and its management. J. Clin. Psychol..

[12] Peris T.S., Rozenman M. (2016). Treatment of scrupulosity in childhood obsessive-compulsive disorder. Clinical Handbook of Obsessive-Compulsive and Related Disorders: A Case-Based Approach to Treating Pediatric and Adult Populations.

[13] Nelson E.A., Abramowitz J.S., Whiteside S.P., Deacon B.J. (2006). Scrupulosity in patients with obsessive–compulsive disorder: Relationship to clinical and cognitive phenomena. J. Anxiety Disord..

[14] Foa E.B., Kozak M.J., Goodman W.K., Hollander E., Jenike M.A., Rasmussen S.A. (1995). DSM-IV field trial: Obsessive-compulsive disorder. Am. J. Psychiatry.

[15] Miller C.H., Hedges D.W. (2008). Scrupulosity disorder: An overview and introductory analysis. J. Anxiety Disord..

[16] Weisner W.M., Riffel P.A. (1960). Scrupulosity: Religion and obsessive compulsive behavior in children. Am. J. Psychiatry.

[17] Reid A.M., Guzick A.G., Fernandez A.G., Deacon B., McNamara J.P., Geffken G.R., McCarty R., Striley C.W. (2018). Exposure therapy for youth with anxiety: Utilization rates and predictors of implementation in a sample of practicing clinicians from across the United States. J. Anxiety Disord..

[18] Huppert J.D., Siev J. (2010). Treating scrupulosity in religious individuals using cognitive-behavioral therapy. Cogn. Behav. Pract..

[19] Schneider S.C., Knott L., Cepeda S.L., Hana L.M., McIngvale E., Goodman W.K., Storch E.A. (2020). Serious negative consequences associated with exposure and response prevention for obsessive-compulsive disorder: A survey of therapist attitudes and experiences. Depress. Anxiety.

[20] Greenberg D., Shefler G. (2008). Ultra-orthodox rabbinic responses to religious obsessive-compulsive disorder. Isr. J. Psychiatry Relat. Sci..

[21] Siev J., Baer L., Minichiello W.E. (2011). Obsessive-compulsive disorder with predominantly scrupulous symptoms: Clinical and religious characteristics. J. Clin. Psychol..

[22] Milgram L., Freeman J., Benito K. (2021). Treating harm avoidance, incompleteness, and disgust in OCD. Brown Univ. Child Adolesc. Behav. Lett..

[23] Purdon C., Cripps E., Faull M., Joseph S., Rowa K. (2007). Development of a Measure of Egodystonicity. J. Cogn. Psychother..

[24] Colón-Bacó E. (2010). The Strength of Religious Beliefs is Important for Subjective Well-Being. Undergrad. Econ. Rev..

[25] Joshi S., Kumari S., Jain M. (2008). Religious Belief and Its Relation to Psychological Well-being. J. Indian Acad. Appl. Psychol..

[26] Simão T.P., Caldeira S., De Carvalho E.C. (2016). The effect of prayer on patients’ health: Systematic literature review. Religions.

[27] Narayanasamy A., Narayanasamy M. (2008). The healing power of prayer and its implications for nursing. Br. J. Nurs..

[28] Whitehouse H. (2024). Rethinking ritual: How rituals made our world and how they could save it. J. R. Anthropol. Inst..

[29] Apffel-Marglin F. (2011). Subversive Spiritualities: How Rituals Enact the World.

[30] Johnson D. (2019). Human Rites: The Power of Rituals, Habits, and Sacraments.

[31] Laing J., Frost W. (2015). Rituals and Traditional Events in the Modern World.

[32] Henderson L.C., Stewart K.E., Koerner N., Rowa K., McCabe R.E., Antony M.M. (2022). Religiosity, spirituality, and obsessive-compulsive disorder-related symptoms in clinical and nonclinical samples. Psychol. Relig. Spiritual..

[33] Rajab A.Z., Elsweedy M.S., Mohamed N.R., Elzahar S.T., Elsayed S.M. (2014). Obsessive-compulsive disorder, an Islamic view. Menoufia Med. J..

[34] Rosmarin D.H., Pirutinsky S., Siev J. (2010). Recognition of scrupulosity and non-religious OCD by Orthodox and non-Orthodox Jews. J. Soc. Clin. Psychol..

[35] Siev J., Huppert J.D., Zuckerman S.E., Abramowitz J.S., McKay D., Storch E.A. (2017). Understanding and treating scrupulosity. The Wiley Handbook of Obsessive Compulsive Disorders.

[36] Guldas F.Z. (2019). Prayer Types and Their Associations with Mental and Psychophysiological Health.

[37] Rosenberg A. (2000). Jewish Liturgy as a Spiritual System: A Prayer-By-Prayer Explanation of the Nature and Meaning of Jewish Worship.

[38] Schachter-Shalomi Z., Segel J. (2013). Jewish with Feeling: A Guide to Meaningful Jewish Practice.

[39] Steinsaltz R.A. (2002). A Guide to Jewish Prayer.

[40] Toprak T.B., Özçelik H.N. (2024). Psychotherapies for the treatment of scrupulosity: A systematic review. Curr. Psychol..

[41] Siev J., Huppert J.D., Storch E.A., Lewin A.B. (2016). Treatment of scrupulosity-related obsessive-compulsive disorder. Clinical Handbook of Obsessive-Compulsive and Related Disorders: A Case-Based Approach to Treating Pediatric and Adult Populations.

[42] Twohig M.P., Abramowitz J.S., Bluett E.J., Fabricant L.E., Jacoby R.J., Morrison K.L., Reuman L., Smith B.M. (2015). Exposure therapy for OCD from an acceptance and commitment therapy (ACT) framework. J. Obs.-Compuls. Relat. Disord..

[43] Wetterneck C.T., Lee E.B., Smith A.H., Hart J.M. (2013). Courage, self-compassion, and values in obsessive-compulsive disorder. J. Context. Behav. Sci..

[44] Papageorgiou D., Karekla M. (2024). Using values interventions to improve exposure therapy engagement in specific phobias. J. Psychother. Integr..

[45] Capel L.K., Twohig M.P. (2025). ACT for OCD: An Example of ACT and Values-Based Exposures. J. Clin. Psychol..

[46] Wicksell R.K. (2007). Values-based exposure and acceptance in the treatment of pediatric chronic pain: From symptom reduction to valued living. Pediatr. Pain Lett..

[47] Lee E.B., Ong C.W., An W., Twohig M.P. (2018). Acceptance and commitment therapy for a case of scrupulosity-related obsessive-compulsive disorder. Bull. Menn. Clin..

[48] Carleton R.N. (2016). Into the unknown: A review and synthesis of contemporary models involving uncertainty. J. Anxiety Disord..

[49] Fergus T.A., Rowatt W.C. (2015). Uncertainty, god, and scrupulosity: Uncertainty salience and priming god concepts interact to cause greater fears of sin. J. Behav. Ther. Exp. Psychiatry.

[50] Boyatzis C.J. (2005). Religious and spiritual development in childhood. Handbook of the Psychology of Religion and Spirituality.

[51] Frankel E. (2017). The Wisdom of Not Knowing: Discovering a Life of Wonder by Embracing Uncertainty.

[52] Greenberg D., Witztum E., Pisante J. (1987). Scrupulosity: Religious attitudes and clinical presentations. Br. J. Med. Psychol..

[53] Spilka B. (2005). Religious practice, ritual, and prayer. Handbook of the Psychology of Religion and Spirituality.

[54] Pruyser P.W. (1968). A Dynamic Psychology of Religion.

[55] Jacobs J.L., Schumaker J.F. (1992). Religious ritual and mental health. Religion and Mental Health.

[56] Sperling J. (2023). The Role of Intolerance of Uncertainty in Treatment for Pediatric Anxiety Disorders and Obsessive-Compulsive Disorder. Evid.-Based Pract. Child Adolesc. Ment. Health.

[57] Craske M.G., Kircanski K., Zelikowsky M., Mystkowski J., Chowdhury N., Baker A. (2008). Optimizing inhibitory learning during exposure therapy. Behav. Res. Ther..

[58] Craske M. (2015). Optimizing Exposure Therapy for Anxiety Disorders: An Inhibitory Learning and Inhibitory Regulation Approach. Verhaltenstherapie.

